# Non-coding accelerated regions underlie polar bear arctic adaptation

**DOI:** 10.21203/rs.3.rs-11224304/v1

**Published:** 2026-10-16

**Authors:** Giada Padovani, Kiley Graim, James Cahill

## Abstract

Polar bears ( *Ursus maritimus)* are an ideal system to understand selective pressures as populations adapt to new environments. Their ecological niche shifted radically from a generalist temperate omnivore to a specialist arctic carnivore, subsisting primarily on seal fat, making polar bears a unique evolutionary model for arctic adaptation. Prior studies of positive selection on protein coding sequences have identified selection on genes involved in cholesterol metabolism. However, selection on non-coding gene regulatory elements, the most common target of positive selection, has not been explored. Here we identified 790 accelerated regions as non-coding targets of positive selection in polar bears. Using two ancient polar bear samples, we were also able to distinguish between recent adaptations and adaptations that were already present in ancient samples. We found significant enrichment for selection on critical gene regulatory systems in polar bears including insulin resistance, inflammation and lipid metabolism, fatty acid degradation, adipogenesis, and pigmentation. Globally, our findings show that selection is acting on pathways that represent important adaptations to the arctic environment such as growth, development, and immunity. This data presents an exciting and novel opportunity to identify the metabolic innovations that allow polar bears to tolerate an extremely high-fat diet, substantially improving knowledge of how species adapt to new climate niches.

## Introduction

Polar bears (*Ursus maritimus*) are an iconic Arctic species that adapted to the Arctic environment in a narrow timeframe since their divergence from brown bears (*Ursus arctos*) 343,000 to 479,000 years ago[1,2]. This adaptation included white coat color, a reduced hibernation regime, and a strictly carnivorous diet with corresponding changes in tooth morphology and cranial structure (Sacco and Van Valkenburgh 2004; Slater et al. 2010). Analysis of positive selection on coding regions of the polar bears' genomes suggest extensive metabolic adaptation to account for their dietary shift, including strong selection on genes involved in cholesterol metabolism and adipose tissue development, but also blood coagulation, heart development, and white coat (Miller et al. 2012; Liu et al. 2014).

The mechanisms of adaptation in polar bears have been a topic of ongoing interest within polar bear literature. There have been studies into selection on coding SNPs (Miller et al. 2012; Liu et al. 2014), and a study of genes' copy number variation (CNV)(Rinker et al. 2019) between polar bears and brown bears which identified nearly 200 genes that displayed species-specific CNV (Rinker et al. 2019). Nearly half of those genes were olfactory receptors with a lower CN in polar bears. Olfactory receptors play a significant role in food or mate detection, and a smaller copy number may indicate a more specific adaptation to the recognition of a narrow variety of prey (Green et al. 2012; Rinker et al. 2019). Fewer copies were also found of genes involved in fatty acid metabolism, also consistent with increased dietary specialization toward hypercarnivory (Rinker et al. 2019).

We present the first study that evaluates the effects of non-coding variations on bear adaptations. This is a major gap in our understanding of arctic adaptation; non-coding cis-regulatory elements (CREs)

comprise the majority of genomic elements under positive selection (Whalen and Pollard 2022). Analysis of non-coding positive selection in humans has found overrepresentation in major regulators of transcription, cell adhesion and brain related functions (Capra et al. 2013; Chu et al. 2020; Whalen and Pollard 2022; Whalen et al. 2023). Recent evidence further points to compensatory interactions between accelerated regions, suggesting that multiple accelerated regions may be responsible for modulation of expression of a single transcript or protein pathway (Whalen et al. 2023).

Detection of positive selection on CREs commonly makes use of an Accelerated Region (AR) approach, that tests for a significant increase in the number of mutations in a CRE relative to the neutral mutation expectation (Pollard et al. 2006). Compared to humans, polar bears pose additional technical challenges. While species trees inferred from the entire genomes of polar bears and brown bears reveal the species to be reciprocally monophyletic, high levels of incomplete lineage sorting (ILS) and admixture result in many gene trees that are not reciprocally monophyletic. A naive application of the Accelerated Region approach would therefore lead to the identification of loci with atypical gene trees from ILS and admixture, rather than loci under positive selection. We developed a population-based approach to the accelerated region approach that allows for better detection of positive selection during what we might term intermediate evolutionary time spans, too ancient for selective sweep-based assays but too recent to be unaffected by ILS and admixture.

In this study we analyze whole genome sequence data from 85 bears to identify Accelerated CREs in polar bears, across 602,629 regions orthologous to human CREs documented by ENCODE. We included two ancient polar bears to distinguish between selection before and after the time of the ancient samples (70–130 kya). We identify 790 polar bears accelerated CREs, that impact a range of key adaptations including lipid metabolism, inflammation response, and pigmentation.

## Results

#### Data Collection and Processing

To identify positive selection in cis-regulatory elements (CREs) in polar bears we begin with the 847,830 proximate and distal enhancers in the ENCODE database used in the Zoonomia multispecies whole genome alignment (Christmas et al. 2023) to identify human-polar bear 1-to-1 orthologs. We require that 1-to-1 orthologs include at least 100-bp of polar bear sequence excluding areas of poor assembly or major structural changes. Finally, to avoid false positive signatures of selection, we identified and removed CREs associated with GC-biased gene conversion (Hubisz et al. 2011). After these filtration steps we retained 602,629 1-to-1 polar bear orthologs of human ENCODE elements suitable for Accelerated Region Analysis.

#### A population-based approach to identifying Accelerated Regions

The presence of incomplete lineage sorting and admixture between polar bears and brown bears confounds a traditional individual-based assessment of accelerated regions. To address this, we developed additional population-based metrics to identify acceleration informative SNPs. We consider a SNP to be informative if it is derived in polar bears and has a high $F_{st}$ between polar bears and brown bears. We define derived alleles not observed in the outgroup American black bears, consistent with an infinite sites assumption (Kimura 1968; Ma et al. 2008). We chose a threshold of $F_{st} > 0.8$ to allow for haplotype calling errors and post-selection gene flow from polar bears into brown bears because of the prevalence of polar bear introgression into brown bears (Cahill et al. 2013; Liu et al. 2014; Cahill et al. 2018; Wang et al. 2022).

We define accelerated regions as ENCODE orthologs that have an excess of acceleration-informative sites (high $F_{st}$, derived) relative to the average across all ENCODE orthologs and applied a Benjamini Hochberg multiple tests correction (Benjamini and Hochberg 2000) following the approach of Hubisz et al (Hubisz et al. 2011). We identified 790 significant ARs ($q<0.05$) within the polar bear lineage. (Figure 1E). AR concentrations peak at 8 ARs/Mb in a polar bear region orthologous to human chromosome 19 (Figure 2A).

**Ancient DNA**

Polar bears' ancient DNA record allows us to define the timing of positive selection events more precisely. Two ancient polar bear samples have been sequenced to high coverage (Laidre et al. 2020; Lan et al. 2021; Laidre et al. 2022; Wang et al. 2022; Sun et al. 2024). These samples date 70-110 thousand years (Wang et al. 2022) and 115-130 thousand years (Lan et al. 2021) respectively. By testing whether derived polar bear alleles predate or postdate the ancient samples, we can determine whether adaptive variants arose before or after time of the ancient individuals (see Figure 4) 454 ARs predate the ancient polar bears, and 44 ARs postdate the ancient polar bears. 271 of these ARs (261 ancient and 10 recent) are also significantly accelerated across the entirety of polar bear evolutionary history. Incomplete overlap between the ARs identified across the whole polar bear evolutionary history and those either pre-dating or post-dating ancient polar bears appears to have multiple causes. First, some elements were likely under positive selection during a time interval that includes the time of the ancient bears. These would not be detected in either the pre- or post-ancient bear tests but would show up overall. The second factor is statistical power; some loci achieve significance FDR <0.05 in one test will fall short of the same significance threshold in another test. However, the combination of this information can provide us with a clearer picture of both the overall trends in polar bear adaptation to the arctic, and how those pressures have changed over time.

**Functional Roles of ARs in Polar Bears**

To determine the likely functional impact of the ARs discovered in this study, we identified the nearest transcription start sites (TSS) upstream and downstream of the human ortholog of each AR with a maximum distance of 1Mb, following both prior methods (McLean et al. 2010) and some of the longest range documented cis-acting enhancers in humans (Lettice et al. 2002; Kurth et al. 2009; Long et al. 2020; Yanchus et al. 2022). Consistent with human data, most polar bear ARs and CREs cluster around the TSS, with the majority of both ARs and CREs falling within 100kb of a TSS (Figure 2B, Table 1). Gene expression prediction approaches have shown greater impacts from sequence changes to enhancers in closer proximity to the TSS. For downstream enrichment analysis, we binned ARs into functional prediction groupings developed for the enhancer functional prediction method ENFORMER (Table 1) (Avsec et al. 2021).

**Table 1** Number of ARs in each distance criteria (column 1) in the whole polar bear lineage (column 2), that arose before the more ancient polar bear sample (column 3), and that arose after the more ancient polar bear sample (column 4).

| Criteria | Polar vs Brown ARs | Pre-Ancient Polar ARs | Post-Ancient Polar ARs | ENCODE orthologs |
|---|---|---|---|---|
| Small (7500bp to 3500bp) | 100 (12.6%) | 56 (12.3%) | 9 (20.5%) | 145,510 (24.1%) |
| Medium (20kbp to 7500bp) | 135 (17.1%) | 75 (16.5%) | 9 (20.5%) | 128,841 (21.4%) |
| Large (100kbp to 20kbp) | 288 (36.5%) | 168 (37.0%) | 15 (34.1%) | 222,381 (36.9%) |
| Extra large (> 100kb) | 267 (33.8%) | 150 (33.0%) | 11 (25.0%) | 105,897 (17.6%) |

We identified 682 genes associated with ARs spanning the entirety of polar bear evolution, 412 genes associated with pre-ancient bear ARs, and 41 genes associated with post-ancient bear ARs. We use these gene lists to test for functional enrichment using g:ProfileR(Kolberg et al. 2023). The pan-polar bear and pre-ancient bear gene lists both produced a large and diverse set of enriched terms. We expected this because a process as complex as arctic adaptation would impact a diverse set of characteristics. However, genes associated with recent ARs produced very few enriched terms. While the shorter timespan suggests a smaller number of selective events, the results are also consistent with a lack of statistical power to perform enrichment analysis with gProfiler, which is plausible given the order of magnitude smaller gene list for recent AR associated genes.

We tested for functional enrichment using 3 major functional pathway databases (KEGG, REACTOME, WikiPathways) (Kanehisa and Goto 2000, Kanehisa et al. 2023; Agrawal et al. 2024; Milacic et al. 2024). Enriched terms are part of four major pathways: Insulin Resistance (KEGG:04931), Pyrimidine Metabolism (KEGG:00240), Signaling by TGFB family members (REAC:R-HSA-9006936), and TGFB receptor signaling (REAC:R-HSA-2173789). While the latter two terms share significant overlap, the Reactome term is larger. Additionally, prior to the ancient bears, we find three significantly enriched terms: Insulin Resistance (KEGG:04931) again, Signal Transduction (REAC:R-HSA-162582), and Neurogenesis regulation in the Olfactory epithelium (WP:WP5265). We observed the greatest enrichment in anatomical structure development (GO:0048856). This captures a substantial subset of the range of known polar bear adaptive processes. TGF-beta signaling is a key mediator of both adipogenesis and associated inflammatory response (Fritsche 2015; Rollins et al. 2015). Likewise, insulin resistance is a major negative consequence of obesity (Hariri and Thibault 2010; Yadav et al. 2011; Wang et al. 2018; Elagizi et al. 2020). Anatomical structure development relates closely to polar bears' physical adaptations, while changes in olfaction are common in adaptation from herbivory to carnivory. These findings confirm the key role of compensatory metabolic adaptations to support hypercarnivory in polar bear evolution.

**Tissues Enriched for Non-coding Positive Selection**

To identify the tissue and organ systems most impacted by polar bears' arctic adaptation, we tested for the expression of AR associated genes in 32 tissue types. Tissue specific gene expression data does not exist for polar bears, and so we harnessed data from 32 human tissue types curated by EMBL-EBI(Moreno et al. 2022). The highest proportion of genes under selection are expressed in pancreas, liver, and heart. Testis, lymph node, and ovary have the least enrichment (Figure S2). The pancreas, liver and heart are all sensitive to disorders deriving from high fat diets (Rinker et al. 2019; Olsen et al. 2021; Rode et al. 2021). These findings support the hypothesis that dietary adaptation is one of the most intensive and far-reaching aspects of polar bears' arctic adaptation.

To further assess whether selection targeted certain systems in specific tissues, we used g:Profiler to identify gene ontology terms enriched in each. Among large pathway databases Reactome, KEGG and WikiPathways, several terms are enriched across >20 of the 32 tissues. The most widely enriched terms (29 tissues) are Neuroinflammation and glutamatergic signaling (WP5083) and Signal Transduction (REAC:R-HSA-162582), followed by pathways involved in TGF-beta receptors, infections, insulin resistance, ammino acid metabolism and AGE/RAGE pathway, with several important genes for inflammation and lipid metabolism. For example, KEGG05200 is linked to MAPK and cAMP signaling pathways, both involved in circadian rhythm regulation and PKA, the activator of PPARa in lipid metabolism that regulates the expression of ACO genes like ACOX2, involved in fatty acid degradation (Table S2).

**Comparison to Protein Coding Positive Selection in Polar Bears**

We tested for overlap between AR associated genes identified in this study and genes under coding positive selection identified in two prior studies (Liu et al. 2014; Sun et al. 2024). Liu et al ranked genes in terms of strength of positive selection but did not define a formal threshold for inclusion of genes as subject to positive selection. Sun et al extended these results, applying a strict set of criteria that yield the eleven loci most likely to be under coding positive selection and compared alleles in modern and ancient polar bears to assess the timing of coding selection. Two of Sun's eleven genes (APOB and LYST) are associated with ARs identified in this study ($p=0.0002$). Coincidentally, APOB and LYST are the most discussed genes in Liu et al, due to their notable associations with Cholesterol metabolism and pigmentation, respectively.

The timing of positive selection on these genes is notable. Both APOB and LYST show ongoing positive selection after the time of the ancient polar bears (Sun et al. 2024), which is consistent with our own findings in non-coding selection. Notably, the AR associated with LYST is post-ancient polar bear, and the AR associated with APOB is significantly accelerated when considering all polar bear history but not when we restrict our analysis to either pre- or post- ancient polar bear. This is consistent with Sun et al's hypothesis that APOB and LYST are targets of very recent positive selection. It therefore calls into question the centrality of these loci to arctic adaptation.

**Obesity related disease resistance - adipogenesis, inflammation and immune response**

We identify a large number of polar bears ARs in regulatory regions associated with metabolic adaptations to hypercarnivory and obesity resistance. These include genes controlling adipogenesis (CD36, NCOA3) and lipid storage and transport (FOXA2, FOXC2, MAL), inflammatory modulation (LRP5, ITIH5, FOXC2, CD36), and cholesterol metabolism (APOB, VLDLR, ABC12, ABC13). Many of these genes function in interconnected pathways and include acceleration both before and after the time of the ancient polar bear samples, indicating selective pressure throughout polar bears evolutionary history (Figure 5).

Polar bears show evidence of positive selection on genes throughout the process of adipogenesis and lipid storage. An AR in close association with the adipogenesis marker gene CD36 (EH38E2566567 -4,095 bp) points to regulatory selection on the production of adipocytes (Gao et al. 2017; Manigandan et al. 2021). Deletion of  NCOA3 (EH38E2117094 -677 bp), reduces body fat and protects against high fat diet induced obesity in mice (Rollins et al. 2015; Bakhshwin et al. 2024). Additional key regulators of lipid storage in strong association with polar bear ARs include FOXC2 (EH38E1835469 -3,272 bp) (Arun 2004; Harms and Seale 2013) and FOXA2 (whole lineage and post-ancient: EH38E2103762 -184,392 bp, pre-ancient: EH38E2103785 -215,924bp) (Perry et al. 2023). Both FOX genes are pioneer transcription factors whose dysregulation is known to promote obesity in mice. Particularly notable within bears, FOXA2 is expression in brown bears in pancreas and regulates proteins implicated in hibernation signaling (Perry et al. 2023) pointing to a potential role in bears seasonal hyperphagia.

Prior research has also pointed to selection for the regulation of cholesterol via APOB (EH38E1979168 -263,381 bp) as a key feature of polar bear adaptation (Liu et al. 2014). Both wild and captive polar bears have the highest concentration of high-density lipoproteins (HDL) reported for Ursidae (Davis and Pagano 2021), allowing them to remove cholesterol more efficiently from the bloodstream. Our dataset also contains ARs associated with several additional cholesterol regulating and processing genes. For example, ABCA12 (EH38E2072666 -131,106 bp) and ABCA13 (EH38E2556053 +699,116 bp) are both involved in cholesterol transport and VLDLR (very low-density lipoprotein receptor) (EH38E2677743 -567,757 bp), is responsible for attaching VLDLs and exchanging triglycerides with tissues (Fujino et al. 2003; Rader 2006).

There is a strong relationship between excess adipocyte generation and chronic inflammation. This association, in turn, is a critical driver of obesity-related negative health outcomes. We find several ARs associated with genes at the nexus of these factors which could point to potential mechanisms by which polar bears evolved resistance mechanisms to obesity related disorders. Low-density lipoprotein-related receptors 5 and 6 (LRP5/6) are highly homologous proteins, crucial in the Wnt pathway. A single missense mutation on LRP6 has been linked to Wnt signaling to coronary artery disease (CAD) and multiple cardiovascular risk factors (Fujino et al. 2003; Mani et al. 2007). LRP6 KO mice gain less weight and show increased thermogenesis (Harms and Seale 2013; Ren et al. 2015). We found one AR associated with LRP5 (EH38E1547874 +42,053 bp). LRP5-deficient mice display hypercholesterolemia and glucose intolerance . Similarly, loss-of-function variants in TBC1D4 are associated with a tenfold increased risk of type 2 diabetes in the Greenlandic Inuit population (Moltke et al. 2014), that also shows positive selection on this gene (Fumagalli et al. 2015)   ITIH5 is associated with 5 ARs in our dataset, the most of any gene (Suppl table S1). ITIH2/5 also interacts with estrogen receptor signaling and is upregulated in inflammatory skin diseases (Hamm et al. 2008; Huth et al. 2015). CD36, mentioned above for its role as adipogenic marker, is also important in lipid transportation during an inflammatory response (Gao et al. 2017; Manigandan et al. 2021). This dual function is supported by acceleration on two regions associated with MAL (EH38E2015958 -1,189 bp and EH38E2015956 -2,873 bp). MAL has a role both in increasing cells surface area in the Golgi apparatus in kidneys and stomach, enhancing apical transport and membrane permeability (Frank 2000; Martín-Belmonte et al. 2001), and in connecting the activation of gpi-anchored proteins (es. cd36) to the external part of the membrane in lymphocytes, especially during an inflammatory response (Martín-Belmonte et al. 2001; Wang et al. 2014). These characteristics indicate that MAL's regulatory regions may have facilitated the adaptation to a hypercarnivorous diet, facilitating or increasing nutrient absorption and delivery through the Golgi apparatus.

#### Cold Induced Adipogenesis

Cold induced adipogenesis, while less clearly linked with human disease, would also have been critical to polar bear evolution, is also under notable selection in polar bears and shares many mechanisms with other features of the adipogenesis/inflammatory system. Increased adiposity is common in animals in cold climates, as a mechanism to enhance insulation and thermogenesis (Derocher et al. 2002; Harms and Seale 2013; Ghaben and Scherer 2019). Cold exposure triggers both the conversion of white to beige adipocytes and the differentiation of brown adipocytes (Magoori et al. 2003; Harms and Seale 2013; Fritsche 2015). We found several ARs that regulate genes involved in cold-induced adipogenesis. TMEM26 (EH38E1471543 +67,627 bp), a beige adipocyte marker (Manigandan et al. 2021). SMAD3 (whole lineage: EH38E1771699 -313,537 bp and pre-ancient: EH38E1771979 -39,915 bp) is an important mediator of this process, mediating TGF-β (EH38E1423186 +211,763 bp and EH38E1423263 +346,760 bp) signaling, which represses adipogenesis by inhibiting the PPARγ complex; its loss promotes thermogenesis and white-to-beige adipocyte conversion (Yadav et al. 2011; Fritsche 2015; Manigandan et al. 2021). Two ARs are associated with EBF2 (whole lineage: EH38E2618610 +106,093 bp and EH38E2618643 +55,564 bp, pre-ancient: EH38E2618643 +55,564 bp), which binds PPARγ to drive brown fat differentiation in small mammalian species in cold environments (Harms and Seale 2013; Rajakumari et al. 2013). ACOX2 and FAM107A are associated with the same AR (EH38E2206447 respectively

-36,532 bp and +53,759 bp). ACOX2 participates in fatty acid oxidation. FAM107A loss enhances fat browning and mitochondrial biogenesis by upregulating PGC-1α, PRDM16, UCP1, and beige-specific genes (Yang and Guan 2013; Zhang et al. 2018; Manigandan et al. 2021). We also identified an AR associated to TBX15 (EH38E1380972 +487076 bp) a transcription factor that directly binds the PRDM16 promoter to regulate adipocyte browning and thermogenesis in response to cold exposure (Sun et al. 2019). Notably, TBX15 was also identified as a target of positive selection in the Greenlandic Inuit population (Fumagalli et al. 2015).

**Pigmentation**

Polar bears' light-colored coat is their most immediately recognizable arctic adaptation. Light coat color is a convergent trait among Arctic predators that plays a pivotal role in hunting success (Blix 2016). Polar bears rely heavily on the camouflage afforded by their light coat to approach their primary prey (ringed seals) (Derocher et al. 2002; Derocher 2012; Blix 2016; Davis and Pagano 2021; Galicia et al. 2021). Although polar bears' fur lacks pigment, their skin is very dark with high levels of melanin production (Derocher 2012). Polar bears have significantly darker skin than brown bears (Leroy et al. 2024), and it has been hypothesized that this is an adaptation to either facilitate heat absorption or provide UV protection (Blix 2016; Carolan et al. 2025;). The conflicting selective pressures for increased melanin in skin and decreased melanin in hair necessitate tissue specific selection mechanisms. Consistent with this expectation, we find ARs associated with pigmentation-related genes: SLC45A2 (EH38E2364645 +1,720 bp), TYRP1 (EH38E2682303 +261,086 bp) and LYST (EH38E1434681 -88,202 bp) (Xu et al. 2013).

SLC45A2 is highly specific to pigmentation. Experimental and genetic studies have found that increased production of SLC45A2 increases melanin production (Dooley et al. 2013; Le et al. 2020; Li et al. 2023). Lower levels or protein coding mutations in the SLC45A2 gene are known to be responsible for albinism type 4 in humans (Rundshagen et al. 2004) and leucism in several other species, including white tigers (Xu et al. 2013). Single cell expression information published on Human Protein Atlas (Moreno et al. 2022) shows SLC45A2 enrichment for pigment cells to an elevated level of specificity (Tau = 0.85, out of a maximum 1). In polar bears, we find an AR 1,720 bp from the SLC45A2 TSS (1,470 bp from the human TSS (The ENCODE Project Consortium 2012; The ENCODE Project Consortium et al. 2020)) (Figure 3A). The enhancer and TSS are on the same contig in the polar bear assembly, and there is strong synteny between humans and polar bears in this region (Armstrong et al. 2020). This extreme proximity and clear synteny allows us a much greater than usual level of confidence that the AR specifically regulates SLC45A2. Despite the genomic proximity of SLC45A2 to AMACR (Figure 3A), only SLC45A2 shows evidence of selection in our dataset. AMACR is a peroxisomal and mitochondrial enzyme that catalyzes the conversion of branched-chain fatty acids from dietary sources, enabling their degradation (Zuo et al. 2022). In humans, the SLC45A2-AMACR gene fusion has been identified as a driving event in several cancer development (Yu et al. 2021; Zuo et al. 2022).

Moreover, chromatin accessibility of this AR in humans is a powerful predictor of SLC45A2 expression. The polar bear AR's human ortholog is significantly enriched in melanocytes (Solé-Boldo et al. 2020; Karlsson et al. 2021) (Figure 3B) and exhibits significant DNase sensitivity in the ENCODE (Moore et al. 2026) database in a melanoma cell line SK-MEL-5 (Figure 3C). In addition, the same database (Moore et al. 2026) shows that H3K4me3 enrichment is the highest in retinal pigment epithelial cells followed by foreskin melanocyte and endocrine pancreas (Figure 3D). Taken together, these lines of evidence for a clear and specific role in melanin production for SLC45A2 and a potent association between the AR"s chromatin accessibility and SLC45A2 gene indicates that this AR likely plays a key role in the polar bear's pigmentation pattern. However, it is not yet clear whether the sequence changes in polar bears are activating (suggesting a role in darkening skin an eye pigmentation) or inactivating underlying the non-melanic hair.

Another notable candidate for involvement in polar bear coat color evolution is TYRP1. TYRP1 is notable because it is one of two genes, along with MCR1 (Hedrick and Ritland 2012), demonstrated to underly coat color variation in an ursid. A single missense mutation in TYRP1 underlies the cinnamon coat color variation in American black bears (Puckett et al. 2023). This mutation leads to decreased melanin production in "cinnamon bears," resulting in a lighter brown coat (Puckett et al. 2023). TYRP1 encodes TRP1 (tyrosinase-related protein) that interacts closely with enzyme tyrosinase (TYR) to catalyze the first steps of melanin formation (Tripathi et al. 1992; Yamaguchi et al. 2007). Mutations in TYRP1 or TYR result in decrease or no production of both eumelanin and pheomelanin. In humans, defects in production or function of TYR can result in oculocutaneous albinism type I or vitiligo, while mutations in TYRP1 result in oculocutaneous albinism type III (Wang and Hebert 2006; Spritz and Hearing 2013). However, this CRE is much more distal, located 261,086 bp from the TSS. This distance is within the observed range of mammalian CRE-TSS distances (Cahill et al. 2021; Martinez-Ara et al. 2022), but our confidence in a functional relationship between a CRE and a TSS should decrease with distance in the absence of additional supporting evidence.

LYST has been the leading candidate locus for positive selection for coat coloration since it was identified as being under coding positive selection in Liu et al 2014 (Liu et al. 2014). LYST is an important regulator of pigmentation in many species (Turner et al. 2024). However, the AR associated with LYST is more recent than the ancient polar bears, as is positive selection on the coding sequence of LYST (Sun et al. 2024). Isotopic evidence indicates that the ancient polar bears had transitioned to a marine diet (Ingólfsson and Wiig 2009). If selection on LYST is responsible for polar bears' white coat, the ancient polar bears (130-70 thousand years ago) probably had brown coats. This is possible; white coats are very rare in non-polar bear ursids (Sato et al. 2011; Hedrick and Ritland 2012; Service et al. 2020), so the earliest polar bear ancestors are unlikely to have had white coats. Ongoing predator-prey conflict and intraspecific competition may have increased the selective advantage of a white coat over time. However, the importance of a white coat to modern polar bears and other arctic predators (Blix 2016), suggests that the more ancient selection of SLC45A2 and TYRP1 is more likely to underly polar bears coat color.

## Discussion

In this study, we identified 790 regions of the polar bear genome under positive selection since their divergence from brown bears. These loci are significantly enriched in metabolic pathways such as insulin resistance and TGF-beta signaling. These adaptations appear to be responses to polar bears' rapid dietary adaptation to an extremely high-fat carnivorous diet. The enrichment of selection on genes expressed in pancreas, heart, and liver further underscores the critical importance of metabolic adaptation for polar bears. Meanwhile, selection on SLC45A2 provides a plausible mechanism for the evolution of polar bears' iconic white coat. We believe these findings significantly advance our understanding of arctic adaptation and provide a myriad of avenues for further research into specific loci.

In identifying the loci targeted by the most common mechanism of adaptive sequence evolution, we provide the most extensive examination of positive selection on polar bears to date. Consistent with previous studies, we find that metabolic and olfactory processes are major targets of selection. We corroborate both selection on the pigmentation associated gene LYST and that this selection is within the last 70,000 years (Liu et al. 2014; Sun et al. 2024). However, the clear advantage of camouflage for successful hunting on sea ice makes such a recent origin of the white coat phenotype unlikely (Thiemann et al. 2008; Blix 2016; Galicia et al. 2021). We consider ancient selection on SLC45A2 and TYRP1 to be much stronger candidates for polar bears coat color adaptations. TYRP1 shows signature of selection both in the whole lineage and in the ancient bears dataset, suggesting earlier selective pressure. The later selection on LYST may represent a refinement after the initial adaptive pressure in addition to corroborating positive selection on the cholesterol metabolism gene APOB, we identify a large group of selection targets involved in regulating obesity-related inflammation. This study therefore is a key step from a handful of important genes to a more systemic, pathway-oriented understanding of polar bears' adaptive history.

A key area of disagreement between the previous studies is whether polar bears underwent more positive selection on more loci than brown bears. The 2014 Liu et al. study of protein coding sequences suggests that polar bears underwent much more positive selection than brown bears. In contrast, Rinker et al 2019 find similar numbers of CNVs between polar bears and brown bears. We find no significant difference between the number of ARs in polar bears and brown bears. While it is possible that polar bears are enriched for protein coding positive selection but not copy number or non-coding selection, another possibility is that the 2014 study systematically underestimates selection in brown bears due to demographic differences. The 2014 study lumps brown bears from both Alaska and Fennoscandia into a single group, despite their long stand divergence (McLellan and Reiner 1994; Edwards et al. 2011; De Jong et al. 2023), would have prevented Liu et al. From identifying most brown bears' population-specific selection. Regardless of which of these hypotheses is correct, our findings indicate that it is the location of ARs that reveal the focus of polar bear selection, not the absolute number of loci under selection.

While we believe that this study provides key context to the process of positive selection on polar bears, it is a first step in understanding the underlying mechanisms of arctic and dietary adaptation. Without direct information on whether a CRE regulates a TSS (Hi-C), the effected tissues (ATAC-Seq), the transcription factors involved (CHiP-Seq), and the impact on gene expression (RNA-Seq), we cannot provide experimental confirmation of these associations. We are fortunate in the case of the SLC45A2 associated AR to have a confluence of human data that reveals an extremely specific functional role of that CRE. Most elements are more pleiotropic impacting more than one tissue type or having less obvious gene expression correlations.

By targeting the main mechanism of positive selection, this study fills an essential gap in polar bear evolutionary history. We have discovered key metabolic and physiological pathways essential for polar bear adaptation. We propose that these findings have the potential to extend beyond evolutionary interest to influence the broader mechanistic understanding of dietary and obesity related diseases. Polar bears' evolutionary approach to resisting these diseases presents a unique lens for additional exploration into drug discovery and therapies to reduce the adverse impacts of obesity in humans.

### Methods

## Data description and processing

We downloaded all publicly available polar bear and brown bear whole genome sequencing data samples with at least 8x coverage and all American black bear samples with at least 4x coverage (Miller et al. 2012; Cahill et al. 2013; Liu et al. 2014; Benazzo et al. 2017; Barlow et al. 2018; Taylor et al. 2018; Laidre et al. 2020; Armstrong et al. 2022; Laidre et al. 2022; Wang et al. 2022) (Table S3) from NCBI using sra fastq-dump (v. 3.0.5)(Leinonen et al. 2011). Our total dataset is comprised of 45 polar bears (43 contemporary and 2 ancient), 30 brown bears, and 10 American black bears. We merged overlapping paired end reads with SeqPrep (https://trace.ncbi.nlm.nih.gov/Traces/sra/sra.cgi?view=software) and mapped with bowtie2 (Langmead and Salzberg 2012) on the polar bear reference genome UrsMar_1.0 (GCA_000687225.1)(Liu et al. 2014). We selected this assembly because its presence in the Zoonomia 241-species alignment (Christmas et al. 2023) allows us to identify polar bear orthologs of human non-coding cis-regulatory elements. We excluded reads with a MapQuality score less than 30, removed duplicates, merged, sorted, and indexed bam files with samtools (v. 1.8) (Li et al. 2009). We assigned read-groups with GATK Picard AddOrReplaceReadGroups (v. 2.25.5)[ https://broadinstitute.github.io/picard/]. Single nucleotide variants were called by using HaplotypeCaller in GATK (v. 4.3.0.0) (McKenna et al. 2010; Poplin et al. 2017).

We annotated orthologs of human non-coding cis-regulatory elements identified by ENCODE (The ENCODE Project Consortium 2012; Luo et al. 2020; The ENCODE Project Consortium et al. 2020) using the Zoonomia 241 species alignment (Christmas et al. 2023). For each ENCODE element we used HalLiftover (v 2.3) (Hickey et al. 2013) to identify homologous polar bear sequences. This methodology tends to produce many polar bear regions per ENCODE element with small gaps between them due to either mapping imperfections or the presence of insertions or deletions. To address this, we merged the inferred polar bear regions separated by less than 30bp with bedtools (v 2.30.0) (Quinlan and Hall 2010).

## Non-coding positive selection detection

The demographic history of polar bears and brown bears including population bottlenecks, incomplete lineage sorting and admixture, poses significant challenges for traditional methods used for detecting non-coding positive selection. To address these challenges, we developed a novel approach based on the accelerated region (AR) approach (Pollard et al. 2006) that integrates large numbers of individuals from each species.

First, to avoid false positive signals of positive selection caused by the recombination driven process GC-biased gene conversion we ran PhastBias (v. 1.4)(Capra et al. 2013) on the branch from terminal branch of the Zoonomia alignment leading to polar bears. We excluded any ENCODE homolog that overlaps a GC-biased region. This is conservative because the branch extends to the common ancestor of all extant bears to polar bears, so it will identify recombination events taking place before the divergence of polar bears and brown bears. However, high levels of incomplete lineage sorting (ILS) and ancient and modern admixture between members of the genus Ursus (Kumar et al. 2017) make polar bear specific GC-bias estimation potentially error prone.

Following the AR model of Pollard et al. (Pollard et al. 2006), we define ARs as regions with an excess of high frequency polar bear specific derived alleles. We identify these in our 85-bear panel as biallelic sites where the polar bear major allele differs from the black bear major allele and $F_{st}$, calculated with VCFtools (v. 0.1.16)(Danecek et al. 2011), between polar bears and brown bears is greater than 0.8. We chose this value rather than fixation ($F_{st}$=1) for two reasons. First, it allows us to detect selection on low frequency standing variation in the polar bear ancestor rather than purely de novo mutations. All non-European sequenced brown bear samples have some introgressed polar bear ancestry, 2-9% depending on the population (Liu et al. 2014; Cahill et al. 2015).

Derived alleles were assessed comparing black bears and polar bears genotypes, while $F_{st}$ were computed with VCFtools (v. 0.1.16)(Danecek et al. 2011). $F_{st}$s were calculated both between modern polar population and ancient polar population, and between modern polar population and modern brown population as well. For whole lineage ARs, we defined informative sites as sites with polar bears derived alleles and $F_{st}$ value between modern polar and brown populations higher than 0.8. This threshold was chosen to avoid the peak due to base conversion at 0.78 (figure S1 suppl). We then counted how many informative sites were present in each region with more than 100bp and corrected p-values with the Benjamini-Hochberg resampling in R (version 4.2.2 (2022-10-31 ucrt)) [https://www.R-project.org/]. The number of regions who had a corrected p-value lower than 0.05 were the ARs. To pinpoint the time of these mutations we used the two ancient polar bears samples. In this case, to define the informative sites, we used the two rules for modern bears adding $F_{st}$ value between modern and ancient polar bears lower than 0.3. This value addresses the small number of ancient samples (4 total alleles) avoiding the result to be driven by case. We also addressed whether informative sites were only present in the most recent bears using the two rules for modern bears with the addition of $F_{st}$ values between modern and ancient polar bears higher than 0.7. (Figure 1 – Table 2)

**Table 2** ARs discovery criteria based on the evolutionary time span. For each time span (whole lineage, ancient bears – before 100,000 years old samples –, and recent bears – after 100,000 years old samples –) we compared polar bears and black bears to assess the derived alleles in polar bears. Then for each time span we selected $F_{st}$ values between polar bears and brown bears greater than 0.8. To differentiate between pre- and post-ancient samples, we added an additional criterion of $F_{st}$ lower than 0.3, and $F_{st}$ greater than 0.7, respectively.

| Timing | Derived alleles in polar bears | $F_{st}$ value (modern polar vs brown) | $F_{st}$ value (modern polar vs ancient polar) |
|---|---|---|---|
| Whole lineage | Yes | > 0.8 | N/A |
| Before ancient samples | Yes | > 0.8 | < 0.3 |
| After ancient samples | Yes | > 0.8 | > 0.7 |

## Enrichment

ARs in human coordinates were used as foreground regions in GREAT (McLean et al. 2010; Tanigawa et al. 2022), with bear mapped ENCODE regions as background. The output (not only enriched genes but full correspondence output) was divided into 3 groups both for regions in modern bears only and for regions shared between modern and ancient bears. The scenarios were chosen depending on the distance of the AR from the gene. The first scenario takes into account genes situated 7500 bp upstream and 3500 bp downstream from the region(Agarwal and Shendure 2020); the second considers genes between 7500 bp and 20 kbp in both directions(Avsec et al. 2021); the third one considers genes between 20kbp and 100kbp in both directions, to integrate information from long-range interactions(Avsec et al. 2021). These lists were used as input in Human Base functional modules detection (Figure 3, Table 1) (Krishnan et al. 2016).

## Tissue specific expression

To evaluate the expression levels, genes associated with ARs were extracted from EMBL-EBI RNA-seq of coding RNA from samples of 122 human individuals representing 32 tissues (Uhlén et al. 2015). The resulting subsets for each tissue were analyzed in gProfiler (Kolberg et al. 2023). KEGG, Reactome and

WikiPathways pathways with a term size number between 20 and 1,000 were selected for each tissue, and the total number of tissues sharing each pathway was computed.

### Declarations

## Author Contribution

J.C. developed the study concept and objectives. All authors designed experiments. G.P. prepared figures and performed analyses. All authors wrote and reviewed the manuscript text.

## Acknowledgement

This study is supported by research funds from the University of Florida as well as funding from (NIH/NCI) R01 CA259306-01.

## Figures

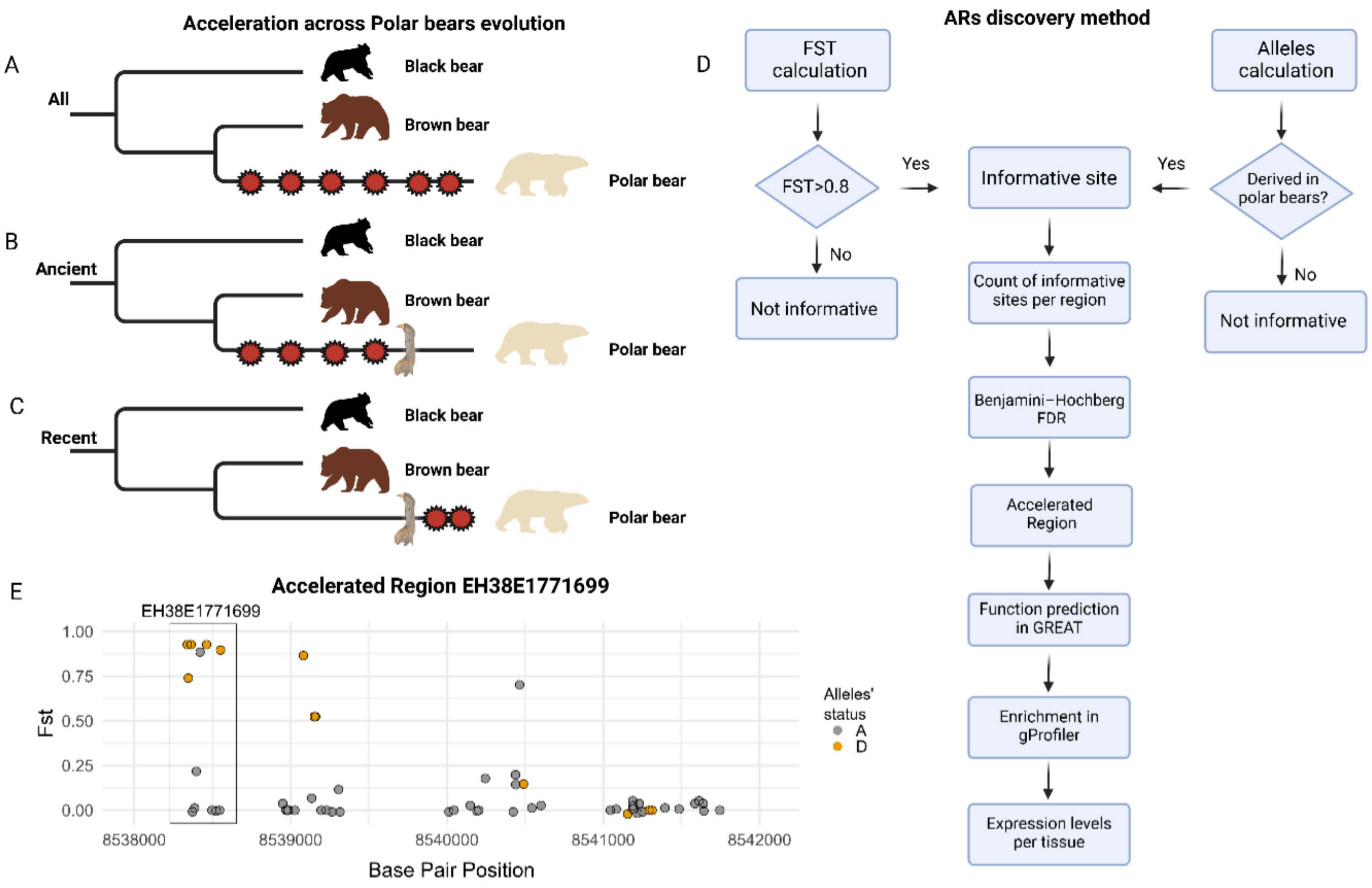

### Figure 1

**A computational approach to discovering noncoding ARs in polar bear evolution.** We conducted three tests for acceleration in polar bear non-coding cis-regulatory elements to determine at what point of the polar bear evolution adaptations arose. A) stylized phylogenetic tree showing the divergence between black bears, brown bears and polar bears and aggregate acceleration across all polar bear evolution. B) Acceleration prior to the 100,000-year-old fossils. C) Acceleration after the 100,000-year-old fossils. D) Flowchart describing our ARs discovery method. E) Example of an AR found considering the number of derived alleles (yellow dots) with high $F_{st}$. Created in BioRender.com

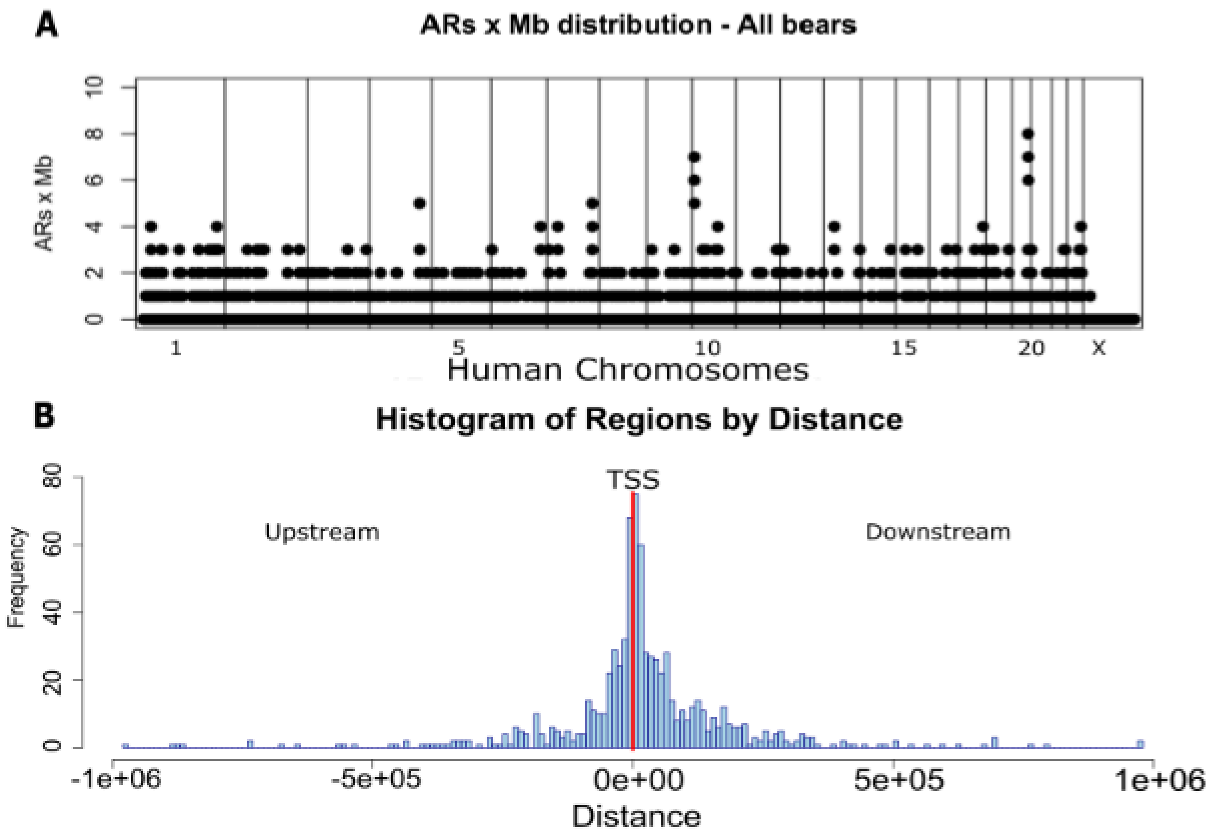

**Figure 2**

**Distribution of accelerated regions and SLC45A2.**

A) ARs distributions in the whole lineage mapped on the human genome. Each dot indicates the number of ARs in a 1-Mb window with windows overlapping one another by 100 kb. Vertical lines demark chromosomes. B) Distribution of ARs in 1 Mb before and after the detected gene.

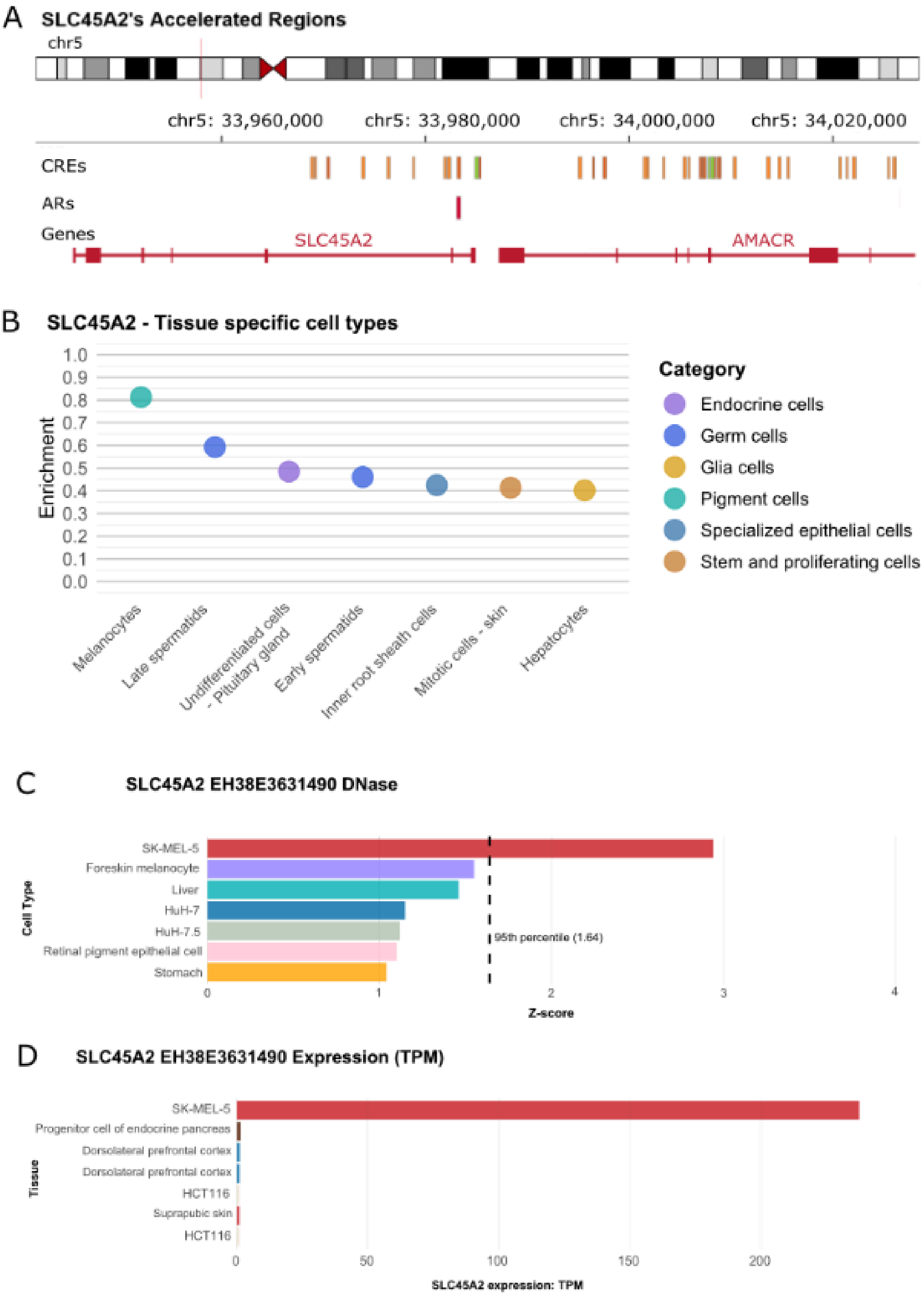

### Figure 3

**Accelerated Region associated with SLC45A2 and its regulatory activity.** A) Representation of CREs in a small section of chromosome 2, and ARs (in red) associated with SLC45A and AMACR. B) Cell type specific enrichment of SLC45A2 expression based on cell types from Human Protein Atlas. C) Chromatin accessibility at AR EH38E3631490 showing SK-MEL-5 as the only significantly expressed cell line in

ENCODE. D) SLC45A2 expression in different cell lines showing SK-MEL-5 as the only significantly expressed.

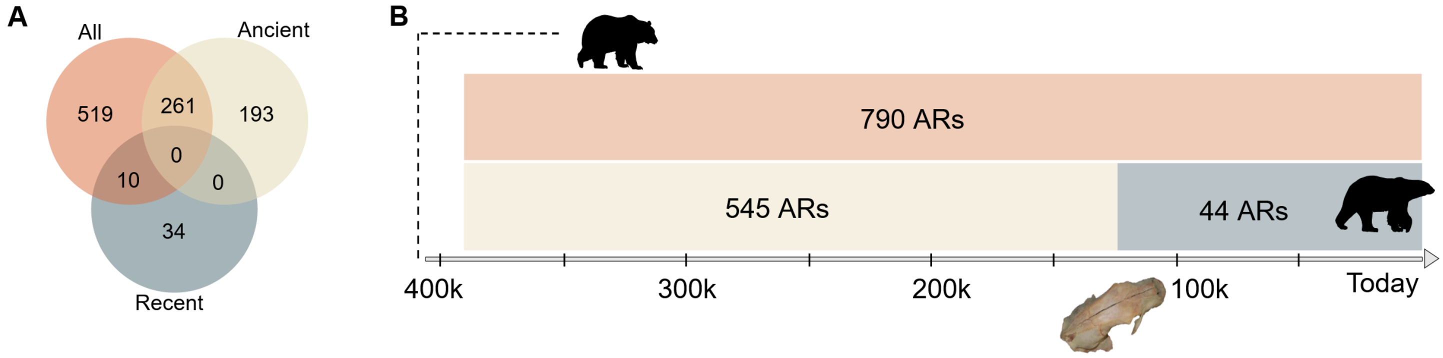

### Figure 4

**Noncoding ARs analysis shows the timing of natural selection.** A) Number of ARs shared and between the whole polar bear lineage (All), before (Ancient) and after (Recent) the 100,000-year-old fossil and B) their location in the evolutionary time span.

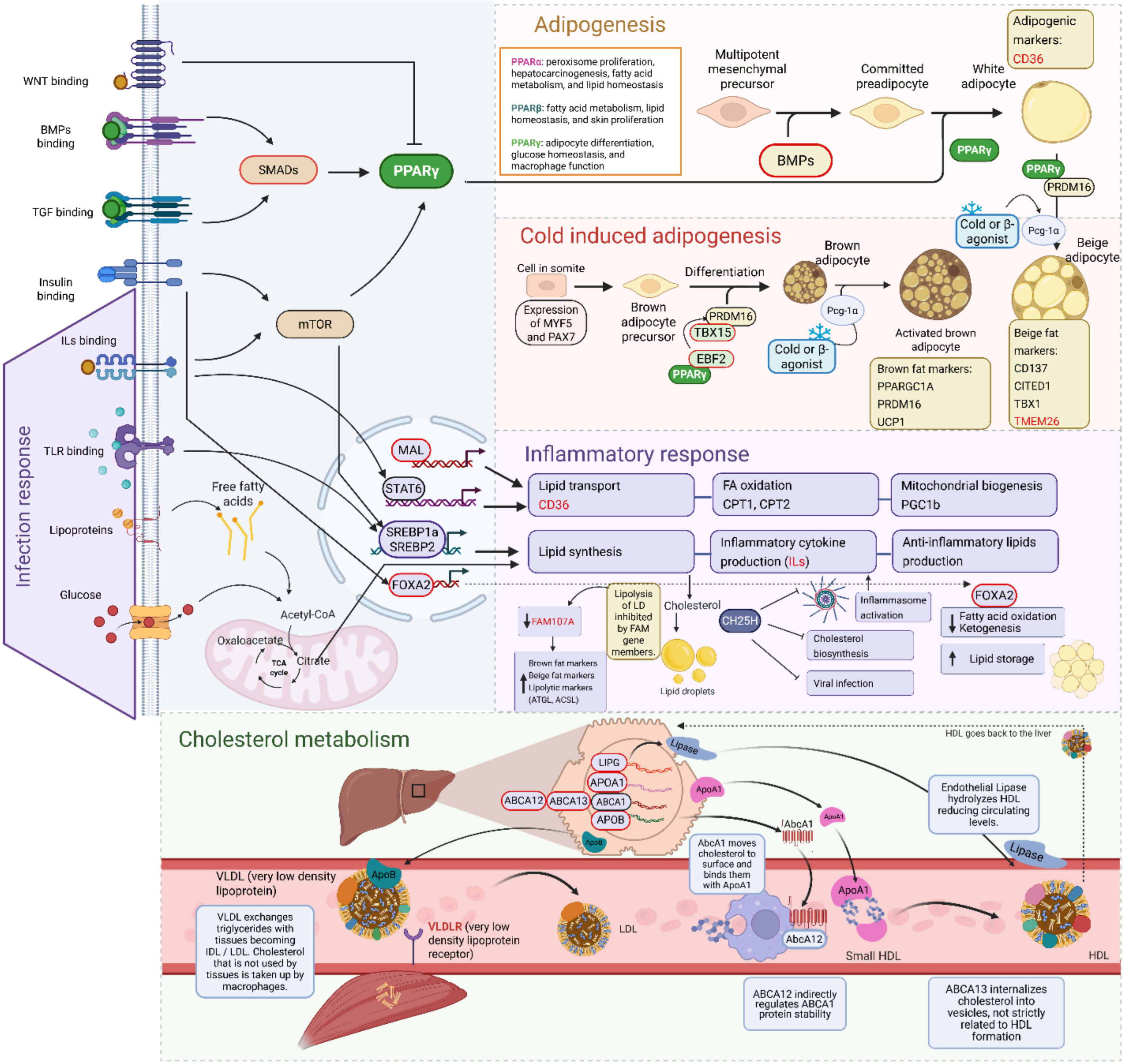

### Figure 5

**Applications to human health.** Schematic representation of the connection between the inflammatory response and adipogenesis. Genes in red or red circled were found in association with ARs in our dataset. Created in BioRender. Padovani, G. (2026) https://BioRender.com/i73o452
